# Commentary: Current Evidence on Traditional Chinese Exercises for Quality of Life in Patients With Essential Hypertension: A Systematic Review and Meta-Analysis

**DOI:** 10.3389/fcvm.2021.801392

**Published:** 2022-01-05

**Authors:** Yifan Gong, Zhibin Liu

**Affiliations:** ^1^The First Clinical Medical School, Henan University of Chinese Medicine, Zhengzhou, China; ^2^Department of AIDS Treatment and Research Center, The First Affiliated Hospital of the Henan University of CM, Zhengzhou, China

**Keywords:** essential hypertension, traditional Chinese exercises, a systematic review, effectiveness, safety

We recently read the article by Song et al. entitled “Current Evidence on Traditional Chinese Exercises for Quality of Life in Patients with Essential Hypertension: A Systematic Review and Meta-Analysis,” published in 2021 (1). The authors reported that Traditional Chinese Exercises (TCE) may be an effective therapy to improve the quality of life (QOL) of patients with essential hypertension, a very encouraging conclusion. However, in our opinion, the authors may be too optimistic with their findings, and we question whether TCE can improve the survival of patients with essential hypertension and inevitably have some concerns.

First, being the most powerful modifiable risk factor for numerous cardiovascular diseases and all-cause mortality (2), hypertension causes millions of deaths from cardiovascular-related causes per year and has a considerable influence on the QOL of patients (3). If we can effectively improve the QOL of patients, it will be of great significance to increase patients' treatment compliance, reduce complications, and improve the prognosis of hypertension. The way to improve hypertension is not only with pharmacological treatment but also with lifestyle improvements, such as using acupuncture and doing exercises (4). Some complementary and alternative therapies have been used and proven to improve clinical symptoms, control blood pressure, and reduce the complications of hypertension (5). For patients with mild hypertension, improving their lifestyle and doing exercise might be effective strategies (6).

Second, TCE has gained popularity worldwide because of its multiple benefits in improving both physical and mental health in patients with chronic diseases (7, 8), which have also been used in the treatment of hypertension (9). TCE is a combination of physical exercise and psychological meditation based on the traditional Chinese medicine theory, rather than simple imitation of limb movements. Therefore, beginners often need to exercise under the guidance of professionals. In this systematic review including 13 original studies, most studies did not describe in detail how patients underwent TCE, and no one assessed the quality of TCE, which leads to the question of whether patients had actually received TCE intervention or had instead performed a kind of limb imitation movement. In other words, if patients received TCE from untrained technicians, the uniformity of TCE would vary greatly, which is very important for the original research results.

Third, among the 13 original studies with older patients, interventions in the treatment group were not limited to TCE, while in the control group only convenient medical management was used. Obviously, the role of those combined methods in the treatment group was ignored by the original researchers, which will directly affect the conclusions of the original study. As it has been reported, the overall quality of the included study is not high. A large sample and high-quality randomized clinical trials were absent from the analysis. Some studies did not describe their sampling method, sample size calculation method, and ethics registration and clinical research registration were not reported. In most included studies, no adverse events were reported, especially in a 1.5-year study, which is obviously very rare. In addition, the original publication bias may seriously affect the authors' systematic evaluation.

In conclusion, we believe that the effectiveness and safety of TCE in improving the QOL of patients with essential hypertension still needs to be confirmed by some high-quality clinical studies. We also hope to see further high-quality systematic evaluations of TCE for other diseases reported in the future.

## Author Contributions

ZL contributed substantially to conception and design and acquisition, analysis and interpretation of data, drafted the article, gave final approval of the version to be published, and agreed to act as guarantor of the work. YG contributed substantially to acquisition analysis and interpretation of data, drafted the article, gave final approval of the version to be published, and agreed to act as guarantor of the work. Both authors contributed to the article and approved the submitted version.

## Funding

This study was supported by the National Special Science and Technology Program on Major Infectious Diseases (Grant Nos. 2017ZX10205502002 and 2017ZX10205502003), Henan Province TCM Research Project (Grant No. 20-21ZYZD03), and Henan Youth Talent Promotion Project (Grant No. 2020HYTP060). The authors are grateful to these funding sources.

## Author Disclaimer

The findings and conclusions of this article are only those of the authors and do not represent the views of the funding sources.

## Conflict of Interest

The authors declare that the research was conducted in the absence of any commercial or financial relationships that could be construed as a potential conflict of interest.

## Publisher's Note

All claims expressed in this article are solely those of the authors and do not necessarily represent those of their affiliated organizations, or those of the publisher, the editors and the reviewers. Any product that may be evaluated in this article, or claim that may be made by its manufacturer, is not guaranteed or endorsed by the publisher.
